# New Functional Motifs for the Targeted Localization of Proteins to the Nucleolus in *Drosophila* and Human Cells

**DOI:** 10.3390/ijms25021230

**Published:** 2024-01-19

**Authors:** Anna A. Ogienko, Mariya O. Korepina, Alexey V. Pindyurin, Evgeniya S. Omelina

**Affiliations:** Institute of Molecular and Cellular Biology, Siberian Branch of the Russian Academy of Sciences, Novosibirsk 630090, Russia

**Keywords:** nucleolus, NoLS, NAD, *Drosophila*

## Abstract

The nucleolus is a significant nuclear organelle that is primarily known for its role in ribosome biogenesis. However, emerging evidence suggests that the nucleolus may have additional functions. Particularly, it is involved in the organization of the three-dimensional structure of the genome. The nucleolus acts as a platform for the clustering of repressed chromatin, although this process is not yet fully understood, especially in the context of *Drosophila*. One way to study the regions of the genome that cluster near the nucleolus in *Drosophila* demands the identification of a reliable nucleolus-localizing signal (NoLS) motif(s) that can highly specifically recruit the protein of interest to the nucleolus. Here, we tested a series of various NoLS motifs from proteins of different species, as well as some of their combinations, for the ability to drive the nucleolar localization of the chimeric H2B-GFP protein. Several short motifs were found to effectively localize the H2B-GFP protein to the nucleolus in over 40% of transfected *Drosophila* S2 cells. Furthermore, it was demonstrated that NoLS motifs derived from *Drosophila* proteins exhibited greater efficiency compared to that of those from other species.

## 1. Introduction

The nucleolus is a specialized organelle within the cell nucleus that plays a crucial role in ribosome biogenesis. It is formed around nucleolus organizer regions (NORs), which consist of clusters of ribosomal DNA (rDNA) genes. Ribosome biogenesis requires a significant amount of energy and is tightly regulated within the cell [1,2,3]. The nucleolus serves as a site for the transcription and processing of ribosomal RNA (rRNA), the main component of ribosomes. The rDNA genes located in the NORs are transcribed into pre-rRNA molecules, which are processed and modified to generate mature rRNA [4]. These mature rRNA molecules, along with ribosomal proteins, are then assembled to form ribosomal subunits [5]. The nucleolus is known for its high transcriptional activity, making it the most active region in the cell nucleus [4]. The high level of nucleolus activity is necessary for meeting the cell’s demand for ribosomes, as protein synthesis is a fundamental process for cell growth and function. In addition to its role in ribosome biogenesis, the nucleolus also has other functions. In human cells, it acts as a “platform” for the organization and clustering of repressed chromatin, which contains specific histone modifications associated with gene silencing [6,7]. In human and mouse embryonic stem cells (ESCs), the nucleolus is involved in regulating gene expression and epigenetic modifications [4]. Furthermore, in human and mouse cells it plays a crucial role in the three-dimensional organization of the genome, contributing to the spatial arrangement of genes and regulatory elements within the nucleus [8]. Overall, the nucleolus is a self-organizing organelle of the nucleus that is responsible for ribosome biogenesis and plays important roles in gene regulation and genome organization. Its prominence and high transcriptional activity highlight its vital contributions to different cellular processes.

**Figure 1:**
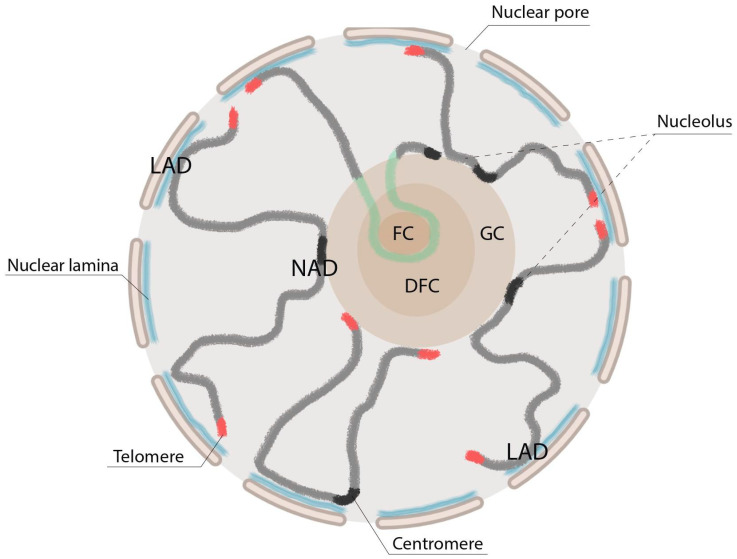
The structure of the nucleus and nucleolus. The major features of the interphase nucleus. The nucleus is surrounded by a nuclear envelope consisting of outer and inner membranes, nuclear pores, and a lamina (shown in blue) located beneath the inner nuclear membrane. Lamina-associated domains (LADs) anchor chromosomes (shown in dark grey) to the lamina at the nucleus periphery, while nucleolus-associated domains (NADs) anchor them to the nucleolus at the center of the nucleus. The nucleolus, a membrane-less organelle, is depicted as a large structure that contains fibrillar centers (FC), dense fibrillar components (DFC), and granular components (GC). The initiation of the transcription of rDNA repeats (shown in green) occurs at the boundary between the FC and DFC. The processing of pre-rRNA occurs in the DFC region, and preribosomal subunit assembly takes place in the GC region. Telomeres (shown in orange) and centromeres (shown in black) tend to cluster at the nuclear periphery and around the nucleolus. This figure was created using BioRender.com.

The nucleolus is surrounded by a shell of chromatin called perinucleolar chromatin. This chromatin is primarily composed of highly condensed heterochromatic DNA that replicates late in the cell cycle [9,10]. The perinucleolar chromatin encompasses individual telomeres, centromeres, and internal chromosome loci [6]. It plays a role in gene silencing by sequestering heterochromatin to the periphery of the nucleolus. This sequestration contributes to the repression of gene expression [11,12,13]. During interphase, the nucleolus can be divided into three compartments: the fibrillar center (FC), the dense fibrillar component (DFC), and the granular component (GC) [14] (Figure 1). The FC borderline is responsible for rDNA transcription, while the DFC and GC are involved in rRNA processing and the assembly of ribosomes, respectively [2,15,16,17]. Studies conducted on human and *Drosophila* cells have provided evidence that a decrease in nucleolar proteins can cause the declusterization of centromeres during interphase and the movement of heterochromatin away from the nucleolar periphery [18,19]. This movement is accompanied by the derepression of centromeric repeats [20]. In specific cases, the depletion of nucleolar proteins such as nucleophosmin 1 (NPM1) in human and mouse normal fibroblasts and cancer cells has been shown to result in rearrangements of perinucleolar heterochromatin [8]. *Drosophila* nucleolar Nucleoplasmin (Nlp) and Modulo (Mod, homolog of the mammalian NCL protein) proteins were shown to play crucial roles in the proper positioning of centromeres close to the nucleolus in the cell. The depletion of either protein led to the unclustering and untethering of centromeres from the nucleolar periphery [19].

The nuclear periphery and the nucleolus were identified as two nuclear landmarks that contribute to repressive chromosome architecture [21]. Nucleolus-associated chromosomal domains (NADs) refer to the regions of the genome that are found near the nucleolus (Figure 1). These regions are typically composed of constitutive and/or facultative heterochromatin, which can be observed in both humans and mice [13,15,22]. NADs contain satellite repeat clusters, inactive rDNA repeats, centromeric regions of most chromosomes, repressed genes with specific histone modifications such as H3K9me2 and H3K9me3, and certain developmentally regulated genes with H3K27me3 modification [6,13,21,22]. Generally, NADs are associated with lower levels of gene expression [3,15].

The sequencing of DNA regions detected around the nucleolus showed that some regions can switch their location between NADs and lamina-associated chromosomal domains (LADs) to maintain gene repression [6,23]. It was mentioned that NADs have higher levels of H3K9me2 and lower levels of H3K27me3, compared to LADs [6,13,21,22]. There is a significant overlap between NADs and LADs [6,24], with approximately 53% of NADs corresponding to LADs and 40% of LADs also being NADs [21]. Two types of NADs have been discovered in mouse embryonic fibroblasts (EFs) and ESCs. Type I NADs overlap with LADs and exhibit characteristics of constitutive heterochromatin, such as late DNA replication, the enrichment of H3K9me3, and low levels of gene expression. On the other hand, type II NADs are exclusively associated with the nucleolus, replicate earlier, display higher gene activity, and are more often enriched with H3K27me3 compared to type I NADs [13,22].

The nuclear lamina’s role in genome organization has been extensively studied due to the identification and characterization of LADs in various cell types [25,26,27,28,29,30,31]. However, research on the nucleolus’ function in this process has been limited, since the nucleolus is a membrane-less liquid-phase organelle [32], and this poses difficulties in developing approaches for identifying NADs [21].

Today, there are several methods for mapping NADs. These methods can be classified into those that rely on the biochemical purification of nucleoli and those that do not [33]. The biochemical purification method involves isolating nucleoli through the ultrasonication of cell nuclei and subsequent sucrose gradient centrifugation steps [34,35]. However, this method has some technical limitations. Heterochromatin, which is more compact and dense, is often resistant to sonication, leading to high noise levels, low information content, and low reproducibility in the resulting NAD profiles. Moreover, it can be challenging to purify nucleoli from certain cell types in complex tissues or organs [21]. Despite these limitations, this method has been used to characterize NADs in cells of various organisms, such as mouse ESCs [13], mouse EFs [22], a few human somatic cell lines [6,7,24], and the plant *Arabidopsis thaliana* [36].

The methods for NAD identification that do not depend on nucleoli purification, such as HiC-rDNA, SPRITE, DNA-seqFISH+, APEX-Seq, and Nucleolar-DamID [7,21,33,37,38,39,40], are more complex and technically demanding than is sequencing the DNA of purified nucleoli; however, they could provide a detailed analysis of NADs that is not biased on chromatin states and that can be performed on every cell type. One of these methods, Nucleolar-DamID, is based on the DNA adenine methyltransferase identification (DamID) approach [41], which was earlier used to identify LADs [25,26,27,28,29,30,31]. However, since the nucleolus is a membrane-free organelle, the application of DamID for the identification of NADs requires further adaptations. To carry this out, it is necessary to find suitable nucleolar localization signal (NoLS) motif(s) that can guide the DNA adenine methyltransferase enzyme to the nucleolus. In an original study [21] conducted to adapt the Nucleolar-DamID method for mapping NADs in mouse ESCs and neuronal progenitor cells, the NoLS sequence RKKRKKK [42,43] was inserted at the C-terminus of the GFP-tagged histone H2B (H2B-GFP-NoLS). This modified histone H2B protein could then bind DNA sequences without motif specificity and localize within the nucleoli. The advantages of the Nucleolar-DamID method include specificity, reproducibility, and broader applicability to different types of cells, including those that make up complex organs/tissues of organisms. In addition, the Nucleolar-DamID method reveals a greater number of NADs compared to methods using the biochemical purification of nucleoli [21]. To date, NADs in *Drosophila* have not been characterized [4]. To adapt the Nucleolar-DamID method to map NADs in *Drosophila*, it is necessary to find suitable NoLS motifs that can guide the DNA adenine methyltransferase enzyme to the nucleolus of *Drosophila* cells.

This study is closely based on the previously described approach [21], and we aimed to identify suitable NoLS motifs in *Drosophila* using the H2B-GFP chimeric protein. The H2B-GFP protein binds to DNA without sequence specificity and can be easily observed in the nucleus and nucleoli. We designed several transgenic constructs that express the histone H2B fused with GFP, along with previously characterized mammalian [43], viral [44], or artificially synthesized [45] NoLS motifs. Next, we created additional constructs that express the chimeric H2B-GFP protein fused with various NoLS motifs from known *Drosophila* nucleolar proteins. These NoLS motifs were predicted using a web tool called Nucleolar Localization Signal Detector (NoD, http://www.compbio.dundee.ac.uk/www-nod, accessed on 3 April 2023) [46,47]. To verify the localization of the H2B-GFP protein fused with different NoLS signals, we performed transient transfection experiments in both *Drosophila* and human cells. The transfected cells were then subjected to immunohistochemical staining using suitable antibodies. As a result, we identified four functional NoLS sequences from different native *Drosophila* nucleolus proteins. When combined, these sequences led to the nucleolar localization of the H2B-GFP protein in over 40% of the transfected *Drosophila* S2 cells.

## 2. Results

### 2.1. Testing of Previously Characterized NoLS Motifs in Drosophila Cell Cultures

**Figure 2:**
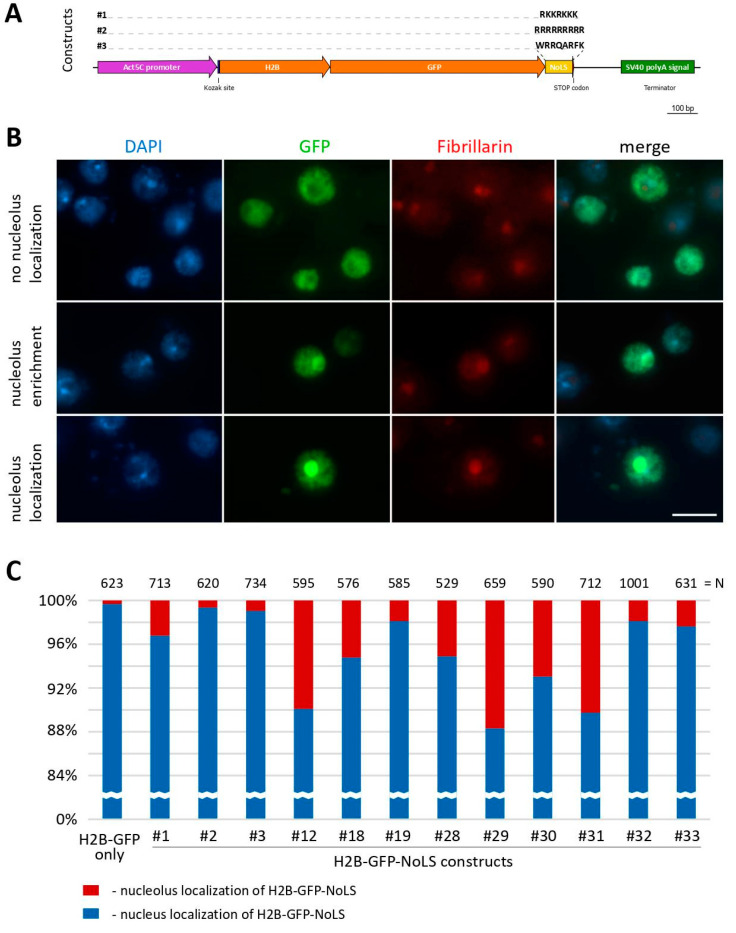
Transfection of *Drosophila* Kc167 cells with plasmids expressing GFP-tagged H2B-NoLS chimeric proteins. (**A**) A scheme of the constructs #1-3 expressing the H2B-GFP chimeric protein with different variants of NoLS motifs (RKKRKKK/RRRRRRRRR/WRRQARFK) used for transient transfection. (**B**) Immunofluorescence images of fixed Kc167 cells 48 h after transfection with plasmid constructs expressing H2B-GFP-NoLS proteins. Transfections with all plasmids showed similar results: no nucleolus localization of the GFP protein; its enrichment in the nucleolus or its clear nucleolar localization. Nuclei of the cells were visualized with DAPI, and nucleoli were detected with antibodies against the nucleolar protein Fibrillarin. Scale bar represents 10 μm. (**C**) Quantitative analysis of the proportion of GFP-positive cells with both obvious nucleolar localization and the enrichment of the GFP signal in the nucleolus. N—the number of cells analyzed. #1–33—the numbers of the plasmid constructs used.

Since no NoLS motifs have been described for *Drosophila*, our aim was to fulfill this gap. For that, an approach described in [21] was utilized. It involved engineering a “nucleolar histone” that possesses two key characteristics: the ability to bind DNA sequences without motif specificity and the capability to localize within nucleoli. We designed transgenic constructs expressing histone H2B fused with GFP and various NoLS motifs attached to the C-terminus of the fusion protein. These motifs included the mammalian RKKRKKK [43], synthetic RRRRRRRRR [45], and viral WRRQARFK [44] sequences (Figure 2). We used *Drosophila* Kc167 cells for pilot experiments, since this cell line had been previously used for the genome-wide mapping of chromatin proteins using the DamID approach [48,49]. After the transient transfection of the Kc167 cells with plasmids expressing the GFP-tagged H2B-NoLS chimeric proteins, we observed a low percentage of cells with nucleolar localization of the GFP protein. For the RKKRKKK, RRRRRRRRR, and WRRQARFK NoLS motifs, only 3.2%, 0.95% and 0.64% of transfected cells showed nucleolar localization of GFP, respectively (Figure 2C). Given the small proportion of cells with obvious nucleolar localization, we also included cells with an enrichmentof the GFP signal in the nucleolus in the analysis (Figure 2B).

### 2.2. NoLS Motifs from Drosophila Nucleolus Proteins

**Figure 3:**
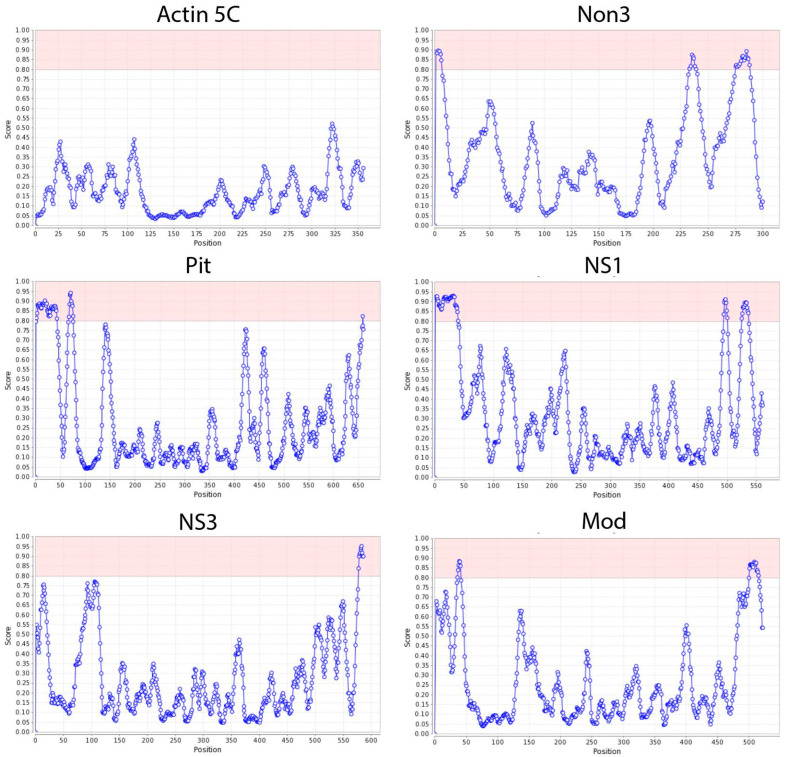
NoLS motifs in *Drosophila* proteins predicted using the NoD web tool (http://www.compbio.dundee.ac.uk/www-nod, accessed on 3 April 2023). Graphs for the nucleolar *Drosophila* proteins Non3, Pit, NS1, NS3, and Mod showing the average NoLS prediction score. The NoLS prediction score for every 20 aa window in the protein is shown on the y-axis. The regions shown in pink are the NoLS candidate segments. The Actin5C protein was used as a non-nucleolus control protein. The position of amino acids in each protein is shown on the x-axis.

Since previously described NoLS motifs such as RKKRKKK/RRRRRRRRR/WRRQARFK were not efficient in localizing the H2B-GFP protein to the nucleolus of Kc167 cells, we analyzed several known *Drosophila* nucleolar proteins (including Mod, Novel nucleolar protein 3 (Non3), Pitchoune (Pit), Nucleostemin 1 (NS1), Nucleostemin 3 (NS3), Jun-related antigen, Fibrillarin, Nucleophosmin, Nucleoplasmin, Nopp140, Nucleostemin 2, and Nop5) using the NoD web tool (http://www.compbio.dundee.ac.uk/www-nod, accessed on 3 April 2023) [46,47] to predict the NoLS motifs in these proteins. Unlike nuclear localization signals (NLSs), NoLSs are less conserved and do not have a universal consensus sequence. Previous attempts to establish a consensus for NoLSs have not been successful [50,51,52]. However, it is known that NoLS motifs usually contain stretches of positively charged amino acid residues such as lysine (K), arginine (R), and histidine (H) [53]. The positive charge is believed to induce protein accumulation in the nucleolus, according to the electrostatic hypothesis [53]. As a result, NoLS motifs were predicted in different regions of the Non3, Pit, NS1, NS3, and Mod proteins (Figure 3, Table 1). At the same time, NoLS motifs were not predicted for the Jun-related antigen, Fibrillarin, Nucleophosmin, Nucleoplasmin, Nopp140, and Nucleostemin 2 proteins. This might be because some proteins that concentrate in the nucleolus lack a well-defined NoLS motif [54,55], or because other NoLS-containing proteins facilitate their recruitment to the nucleolus [42,56].

### 2.3. NoLS Motifs from Drosophila Nucleolus Proteins

**Figure 4:**
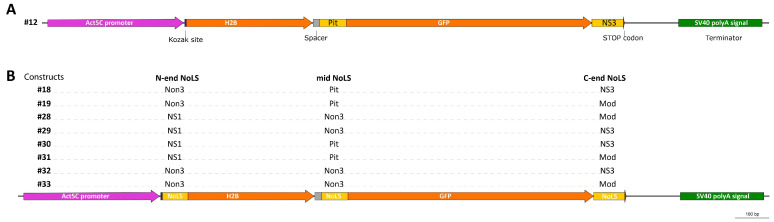
A scheme representing plasmid constructs expressing H2B-GFP chimeric proteins with different variants of predicted NoLS motifs (shown as yellow rectangles). (**A**) Construct #12 carries two NoLS motifs, in the middle and at the C-terminal end of the H2B-GFP chimeric protein. (**B**) Constructs #18–33 carry three NoLS motifs, at the N-terminal end, in the middle, and at the C-terminal end of H2B-GFP.

We designed nine different plasmid constructs that express histone H2B fused with GFP and at least two NoLS motifs predicted by the NoD web tool (Table 1, Figure 4). These NoLS motifs were taken from the N-terminal and C-terminal ends of native nucleolar proteins and placed accordingly in the constructs. The middle-region NoLS motifs of the native nucleolar proteins were inserted between the sequences of the H2B and GFP proteins.

**Figure 5:**
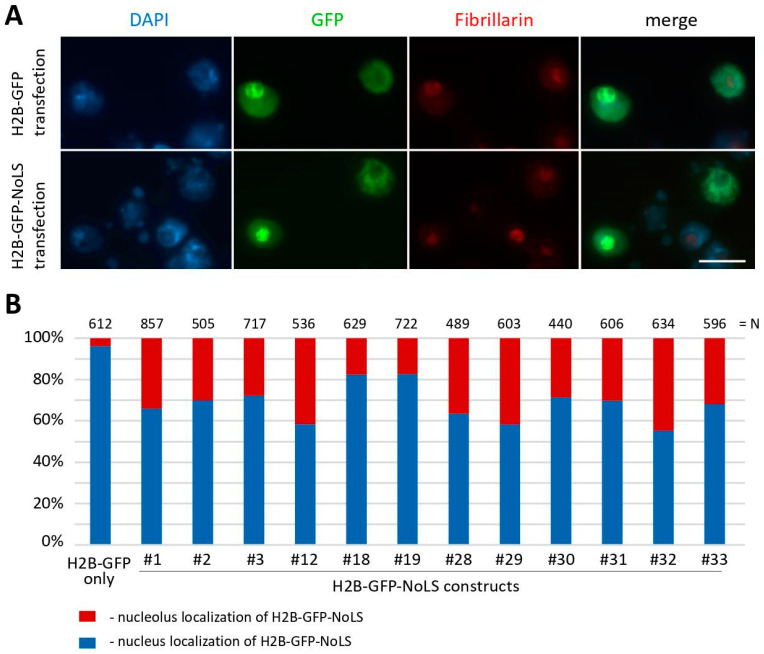
Transfection of *Drosophila* S2 cells with plasmids expressing various GFP-tagged H2B-NoLS proteins. (**A**) Immunofluorescence images of fixed S2 cells 48 h after transfection with plasmid constructs expressing H2B-GFP-NoLS proteins. Cells with bright green spots show obvious nucleolar localization of GFP. Nuclei of the cells were visualized with DAPI, and nucleoli were detected with antibodies against the nucleolar protein Fibrillarin. Scale bar represents 10 μm. (**B**) Quantitative analysis of the proportion of GFP-positive cells with both obvious nucleolar localization and the enrichment of the GFP signal in the nucleolus. N—the number of cells analyzed. #1–33—the numbers of the plasmid constructs used.

We tested these constructs via the transient transfection of *Drosophila* Kc167 cells, and found that three plasmids (#12, #29, and #31) showed better but not sufficient nucleolar localization of the GFP protein (9.9%, 11.7%, and 10.25% of transfected cells, respectively), as shown in Figure 2C. We also performed transient transfection of *Drosophila* S2 cells with the same plasmid constructs. Surprisingly, in S2 cells, all the constructs showed nucleolar localization in a significant proportion of transfected cells, as depicted in Figure 5. Unlike Kc167 cells, S2 cells exhibited more prominent nucleolar localization of the GFP protein. Again, three plasmids (#12, #29, and #32) showed higher nucleolar localization of H2B-GFP in S2 cells (41.6%, 41.8%, and 44.8% of transfected cells, respectively), as shown in Figure 5B. It is worth noting that the H2B-GFP construct carrying the previously described NoLS RKKRKKK (#1) also showed substantial nucleolar localization in S2 cells (34.0% of transfected cells) (Figure 5B) unlike in Kc167 cells (3.2% of transfected cells) (Figure 2C).

**Figure 6:**
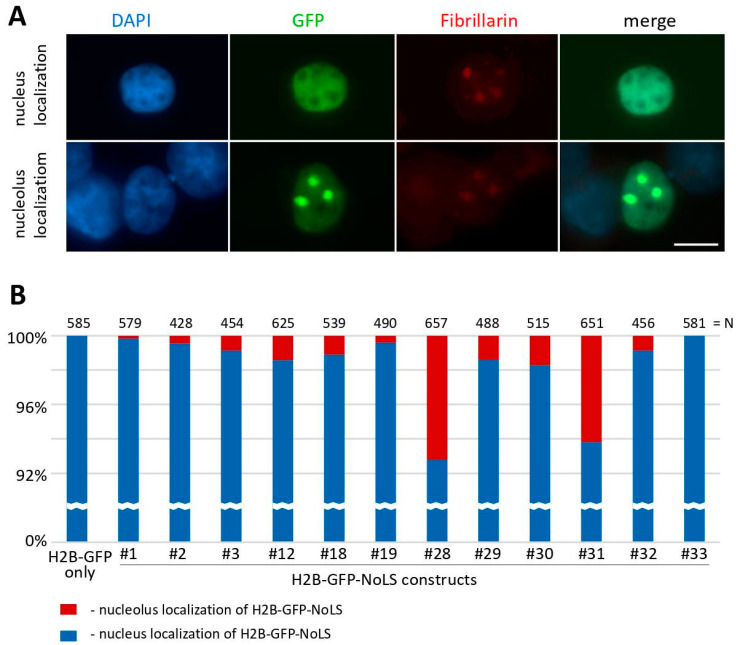
Transfection of human HEK293T cells with plasmids expressing the GFP-tagged H2B-NoLS proteins. (**A**) Immunofluorescence images of fixed HEK293T cells 48 h after transfection with plasmid constructs expressing H2B-GFP-NoLS proteins. Cells with bright green spots show obvious nucleolar localization of H2B-GFP. Nuclei of the cells were visualized with DAPI, and nucleoli were detected with antibodies against the nucleolar protein Fibrillarin. Scale bar represents 10 μm. (**B**) Quantitative analysis of the proportion of GFP-positive cells with both obvious nucleolar localization and the enrichment of the GFP signal in the nucleolus. N—the number of cells analyzed. #1–33—the numbers of the plasmid constructs used.

In the previous study [21], live cell imaging was used to investigate the localization of the GFP-tagged H2B-NoLS (the RKKRKKK NoLS) protein in NIH3T3 cells. It was found that this protein showed prominent and preferential localization in nucleoli compared to its uniform distribution throughout the nucleus of the H2B-GFP protein without NoLS. To test the species specificity of NoLS motifs identified in *Drosophila* nucleolar proteins, we transiently transfected human HEK293T cells with all our plasmid constructs, as shown in Figure 6. However, we observed that the RKKRKKK NoLS does not provide nucleolar localization in HEK293T cells, as only 0.17% of transfected cells exhibited a GFP signal in nucleoli (see construct #1 in Figure 6B). The usage of other variants of NoLS motifs resulted in a higher percentage of nucleolar localization of the GFP protein in HEK293T cells. For example, constructs #28 and #31 showed 7.2% and 6.18% transfected cells with nucleolar localization of the GFP-tagged H2B-NoLS protein, respectively.

## 3. Discussion

The nucleolus, functioning as a ribosome factory in the nucleus of eukaryotic cells, plays a crucial role in sustaining the protein synthesis machinery [4]. Recent research suggests that the nucleolus may also have additional functions beyond ribosome biogenesis. One intriguing role is its involvement in organizing the 3D genome structure. However, our current understanding of this aspect of genome compartmentalization in the cell nucleus is still limited, especially in the case of *Drosophila*. We aimed to advance our understanding of this problem. To carry this out, it is crucial to identify specific NoLS motifs that work well for *Drosophila*. NoLS motifs are short amino acid sequences that are responsible for targeting proteins to the nucleolus. These sequences have been identified and characterized in various organisms, including humans, yeast, and other model organisms. However, NoLS motifs for *Drosophila* have not been extensively studied and described compared to those for other species.

In this study, we conducted an extensive series of experiments to determine the most effective NoLS motifs for targeting the H2B-GFP chimeric protein to the nucleolus in *Drosophila* Kc167 and S2 cell lines. We found that previously described mammalian (RKKRKKK) [43], viral (WRRQARFK) [44], and artificially synthesized (RRRRRRRRR) [45] NoLS motifs show low efficiency in Kc167 cells. No more than 3% of transfected Kc167 cells showed nucleolar localization of the GFP protein. To increase the number of cells with nucleolar localization of the GFP protein, we additionally designed nine constructs encoding H2B-GFP tagged with NoLS motifs predicted in native *Drosophila* nucleolus proteins. As a result, these constructs proved to be somewhat more effective. However, the increase in protein nucleolar localization was modest, with the best constructs showing around 5% efficiency in Kc167 cells. The best percentage of GFP nucleolar localization was provided by constructs ##12, 29 and 31 with two or three NoLS motifs from the Pit, NS1, NS3, Non3, and Mod proteins.

In contrast, about 34% of S2 cells showed nucleolar localization of H2B-GFP tagged with the RKKRKKK NoLS (construct #1). The most effective constructs with prominent nucleolar localization of GFP in S2 cells were ##12, 29 and 32, carrying two or three NoLS motifs from the nucleolar proteins Pit, NS1, NS3, and Non3. Thus, the highest level of nucleolar localization was for constructs #12 and #29 both in Kc167 and S2 cell lines. Accordingly, the most effective *Drosophila* NoLS motifs are from the middle region of the Pit protein (EDLYKQARKQPKQLKVGKKKNISTDA), the C-terminal end of the NS3 protein (GNDPAAKPWRHVKKERREKLRKKFSHLDEH), the N-terminal end of the NS1 protein (MALKRLKTKKSKRLTGRLKHKIEKKVRDHNKKERRAAKKNPKKGSKKQKLIQIPNICPF), and the middle region of the Non3 protein (EDLYKQARKQPKQLKVGKKKNISTDA).

Interestingly, analyzed NoLS motifs have different efficiencies of nucleolar localization of the GFP protein not only in cells of different species (for example, in *Drosophila* S2 and human HEK293T cells), but also between different cell lines of the same species (*Drosophila* S2 and Kc167). The variability in the efficiency of these NoLS motifs between different cell types, even within the same species, can result from several factors. First of all, different cell types can express varying levels of receptors or transporters responsible for the recognition of these signals. The cellular machinery that interprets localization signals may also differ, which can lead to variability in protein targeting among cell lines. The nucleolus is a dynamic organelle without a membrane, and its architecture could differ between cell types [57,58]. The size and number of nucleoli could influence the localization efficiency of proteins with NoLS motifs. The efficiency of protein localization can depend on the presence and availability of interaction partners within the cell. If other proteins that facilitate localization to the nucleolus are expressed at different levels, this could influence NoLS efficiency. Additionally, proteins undergo various post-translational modifications that can affect their localization. If enzymes providing such modifications are differently expressed or active in different cell lines, this could affect the nucleolar localization of proteins. When comparing across species, such as between *Drosophila* S2 cells and human HEK293T cells, there are fundamental differences in the sequence and structure of the proteins and nucleic acids involved in nucleolar localization, which can lead to differences in the efficiency. Thus, this phenomenon points to the complex nature of cellular processes, the specificity of certain sequences and their function, particularly with regard to protein localization within cells.

We suggest that these results are quite significant for experiments where efficient nucleolar localization is desired. Here, we provide a refined toolkit of NoLS motifs that can be utilized and potentially combined or further optimized for increased efficiency of nucleolar targeting in *Drosophila* or even extrapolated for use in other species.

## 4. Materials and Methods

### 4.1. Isolation of total RNA, Reverse Transcription

Total RNA was isolated from 15 larvae of Oregon *R* flies (modENCODE stock #25211) using RNAzol RT (Molecular Research Center, Cincinnati, OH, USA) in accordance with the manufacturer’s recommendations. The isolated RNA was incubated with 3 U of DNase I (ThermoFisher Scientific, Waltham, MA, USA) for 30 min at 37 °C. The CleanRNA Standard kit (Evrogen, Moscow, Russia) was used for RNA purification. Briefly, 2 μg of total RNA was mixed with 1 μL of the 50 mM oligo(dT)_20_ primer in a total volume of 13.5 μL, and the mixture was incubated for 5 min at 65 °C. The reverse transcription reaction was carried out in a volume of 20 μL with the following components: 13.5 μL of RNA template with annealed primers, 4 μL of 5× RT buffer (ThermoFisher Scientific), 1 μL of 10 mM dNTP, 1 μL of RNaseOUT (ThermoFisher Scientific), and 100 U RevertAid reverse transcriptase (ThermoFisher Scientific). The mixture was incubated for 60 min at 42 °C, and the enzyme was inactivated for 10 min at 70 °C.

### 4.2. Isolation of Genomic DNA

Isolation of genomic DNA from 30–50 anesthetized *Drosophila* adult flies (line #25211, modENCODE) was performed in accordance with [59].

### 4.3. Generation of the p-p.Actin5C-H2B-GFP Constructs Encoding Proteins with the RKKRKKK, RRRRRRRRR and WRRQARFK NoLS Motifs

The plasmid p-p.Actin5C-H2B-GFP [60] was hydrolyzed at EcoRI and XbaI restriction sites. The insertions encoding the RKKRKKK [43], RRRRRRRRR [45] and WRRQARFK [44] NoLS motifs were amplified using the primers GFP-EcoRI-fwd and GFP-RKKRKKK-NoLS-XbaI-rev, GFP-R9-NoLS-XbaI-rev and GFP-WRRQARFK-NoLS-XbaI-rev (Table 2), respectively. To 50 μL of the reaction mixture, 1 ng of the plasmid p-p.Actin5C-H2B-GFP template, 0.5 μL of Phusion polymerase (ThermoFisher Scientific), 1 μL of 10 μM primers, and dNTPs at 0.2 mM were added. The PCR conditions were as follows: 98 °C for 30 s, 35 cycles of 98 °C for 10 s, 65 °C for 10 s, 72 °C for 30 s, and incubation for 10 min at 72 °C.

### 4.4. Generation of the p-p.Actin5C-H2B-GFP Constructs Encoding Proteins with Different Predicted Drosophila NoLS Motifs 

The plasmids p-p12.Actin5C-H2B-mid_pitch-GFP-C-end_NS3, p-p18.Actin5C-N-end_Non3-H2B-mid_pitch-GFP-C-end_NS3, p-p28.Actin5C-N-end_NS1-H2B-mid_Non3-GFP-C-end_Mod, p-p29.Actin5C-N-end_NS1-H2B-mid_Non3-GFP-C-end_NS3, p-p19.Actin5C-N-end_Non3-H2B-mid_pitch-GFP-C-end_Mod, p-p30.Actin5C-N-end_NS1-H2B-mid_pitch-GFP-C-end_NS3, p-p31.Actin5C-N-end_NS1-H2B-mid_pitch-GFP-C-end_Mod, and p-p32.Actin5C-N-end_Non3-H2B-mid_Non3-GFP-C-end_NS3 were generated using the Gibson cloning method. The plasmid p-p.Actin5C-H2B-GFP [60] was hydrolyzed at KpnI and XbaI restriction sites. The fragments encoding NoLS motifs at the N-terminal end and other NoLS motifs were amplified using cDNA and genomic DNA from Oregon *R* flies (modENCODE stock #25211) as a DNA template, respectively. Other DNA fragments were amplified from the plasmid p-p.Actin5C-H2B-GFP as a DNA template.

The primers Fr1_for_Kozak_H2B and Fr1_rev_pitch, Fr2_for_pitch and Fr2_rev_pitch, Fr3_for_pitch_EGFP and Fr2_rev_EGFP_NS3, and Fr3_for_NS3 and Fr3_rev_NS3 were used to amplify the inserts to assemble the construct p-p12.Actin5C-H2B-mid_pitch-GFP-C-end_NS3.

The primers Fr1_for_Non3 and Fr1_rev_Non3_H2B, Fr2_for_Non3_H2B and Fr1_rev_pitch, Fr2_for_pitch and Fr2_rev_pitch, Fr3_for_pitch_EGFP and Fr2_rev_EGFP_NS3, and Fr3_for_NS3 and Fr3_rev_NS3 were used to amplify the inserts to assemble the construct p-p18.Actin5C-N-end_Non3-H2B-mid_pitch-GFP-C-end_NS3.

The primers Fr1_for_NS1 and Fr1_rev_NS1, Fr2_for_NS1_H2B and Fr1_rev_Non3, Fr2_for_Non3 and Fr2_rev_Non3, Fr3_for_Non3_EGFP and Fr2_rev_EGFP_mod, and Fr3_for_mod and Fr3_rev_mod were used to amplify the inserts to assemble the construct p-p28.Actin5C-N-end_NS1-H2B-mid_Non3-GFP-C-end_Mod.

The primers Fr1_for_NS1 and Fr1_rev_NS1, Fr2_for_NS1_H2B and Fr1_rev_Non3, Fr2_for_Non3 and Fr2_rev_Non3, Fr3_for_Non3_EGFP and Fr2_rev_EGFP_NS3, and Fr3_for_NS3 and Fr3_rev_NS3 were used to amplify the inserts to assemble the construct p-p29.Actin5C-N-end_NS1-H2B-mid_Non3-GFP-C-end_NS3.

The primers Fr1_for_Non3 and Fr1_rev_Non3_H2B, Fr2_for_Non3_H2B and Fr1_rev_pitch, Fr2_for_pitch and Fr2_rev_pitch, Fr3_for_pitch_EGFP and Fr2_rev_EGFP_mod, and Fr3_for_mod and Fr3_rev_mod were used to amplify the inserts to assemble the construct p-p19.Actin5C-N-end_Non3-H2B-mid_pitch-GFP-C-end_Mod.

The primers Fr1_for_NS1 and Fr1_rev_NS1, Fr2_for_NS1_H2B and Fr1_rev_pitch, Fr2_for_pitch and Fr2_rev_pitch, Fr3_for_pitch_EGFP and Fr2_rev_EGFP_NS3, and Fr3_for_NS3 and Fr3_rev_NS3 were used to amplify the inserts to assemble the construct p-p30.Actin5C-N-end_NS1-H2B-mid_pitch-GFP-C-end_NS3.

The primers Fr1_for_NS1 and Fr1_rev_NS1, Fr2_for_NS1_H2B and Fr1_rev_pitch, Fr2_for_pitch and Fr2_rev_pitch, Fr3_for_pitch_EGFP and Fr2_rev_EGFP_mod, and Fr3_for_mod and Fr3_rev_mod were used to amplify the inserts to assemble the construct p-p31.Actin5C-N-end_NS1-H2B-mid_pitch-GFP-C-end_Mod.

The primers Fr1_for_Non3 and Fr1_rev_Non3_H2B, Fr2_for_Non3_H2B and Fr1_rev_Non3, Fr2_for_Non3 and Fr2_rev_Non3, Fr3_for_Non3_EGFP and Fr2_rev_EGFP_NS3, and Fr3_for_NS3 and Fr3_rev_NS3 were used to amplify the inserts to assemble the construct p-p32.Actin5C-N-end_Non3-H2B-mid_Non3-GFP-C-end_NS3.

To 50 μL of the reaction mixture, 1 ng of DNA template, 0.5 μL of Phusion polymerase (ThermoFisher Scientific), 1 μL of 10 μM primers, and dNTPs at 0.2 mM were added. The PCR conditions were as follows: 98 °C for 30 s, 35 cycles of 98 °C for 10 s, 62 °C for 10 s, 72 °C for 1 min, and incubation for 10 min at 72 °C. After purification, 50 ng of “vector” and a two-fold molar excess of each “insert” were mixed with 10 μL of 2 × NEBuilder HiFi DNA Assembly Master (NEB, Ipswich, MA, USA) in a total volume of 20 μL.

### 4.5. Transfection of Kc167, S2 and HEK293T Cells

Twenty-four hours before transfection, Kc167, S2, and HEK293T cells were seeded into a 6-well plate at a concentration of 5 × 10^5^ cells per well in 2 mL of Shields and Sang M3 Insect Medium (Sigma-Aldrich, Burlington, MA, USA) supplemented with 10% heat-inactivated bovine serum or IMDM medium (Gibco, Grand Island, NY, YSA) supplemented with 10% bovine serum, respectively. Cells were transfected with plasmid DNA (3 μg) using PEI-Transferrinfection Kit (ThermoFisher Scientific) in accordance with the manufacturer’s recommendations. After transfection, the cells were cultured for 48–72 h.

### 4.6. Immunofluorescence Staining and Microscope Analysis

For microscopic analysis, cells were washed in phosphate-buffered saline (PBS) and fixed in PBS containing 3.7% formaldehyde (Merck, Rahway, NJ, USA) for 10 min. Cells were resuspended in 500 µL of PBS, and cytocentrifuged on clean slides (using a Cytospin 4 Cytocentrifuge, Thermo Fisher Scientific, at 900 rpm for 4 min). Slides were then immersed in liquid nitrogen for 5 min, transferred to PBS containing 0.1% TritonX-100 for 30 min, and then transferred to PBS containing 3% BSA (AppliChem, Darmstadt, Germany) for 30 min. The slides were immunostained using the following primary antibodies, all diluted in a 1:1 mixture of PBT and 0.3% BSA: chicken anti-GFP (1:200, Thermo Fisher Scientific, PA1-9533) and mouse anti-Fibrillarin (1:200, Thermo Fisher Scientific, MA1-22000). Primary antibodies were detected via incubation for 1 h with Alexa Fluor 488-conjugated goat anti-chicken IgG (1:300, Thermo Fisher Scientific, A11039) and FITC-conjugated goat anti-mouse IgG (1:40, Sigma-Aldrich, Burlington, MA, USA, F8264). Slides were then mounted in Vectashield antifade mounting medium containing 4.6-diamidino-2-phenylindole (DAPI; Vector Laboratories, Newark, CA, USA). Images of fixed cells were captured using Zeiss Axio Imager M2 equipped with an EC Plan-Neofluar 100×/1.30 oil lens (Carl Zeiss Microscopy, Oberkochen, Germany) and with an AxioCam 506 mono (D) camera.

## Figures and Tables

**Table 1:** Amino acid sequences of predicted *Drosophila* NoLS motifs. The NoLS motifs used in the study are underlined.

Protein	N-End NoLS	Middle NoLS	C-End NoLS
Novel nucleolar protein 3 (Non3)	MSLLRIRKPKTRKGKKVLLAREPQL (positions 1–25)	EDLYKQARKQPKQLKVGKKKNISTDA (positions 233–258)	SIQTRRVKALRKTPEEKKENRQRKKVALKAAAA (positions 275–307)
Pitchoune (Pit)	SIREKLLMKKIVKREKMKKELSQKKGNKNAQKQEPPKQNGNKPSKKPEKLSKKHVAKDEDD (positions 2–62)	DFQEAPLPKKKQQKQPPKKQQIQVANSD (positions 67–94)	GSASKQRHFKQVNRDQAKKF (positions 659–678)
Nucleostemin 1 (NS1)	MALKRLKTKKSKRLTGRLKHKIEKKVRDHNKKERRAAKKNPKKGSKKQKLIQIPNICPF (positions 1–59)	VIDEKEKPAKGRKRKLDEEKEKVDPS (positions 494–519)	NQSLNKGIKQMQKLKKKQNVRNEKKISKITD (positions 525–555)
Nucleostemin 3 (NS3)	-	-	GNDPAAKPWRHVKKERREKLRKKFSHLDEH (positions 577–606)
Modulo (Mod)	ETVVPQSPSKKSRKQPVKEVPQFSE (positions 36–60)	-	IGQTRAPRKFQKDTKPNFGKKPFNKRPAQENGGK (positions 501–534)

**Table 2:** Primers used in the study.

Primer Name	Sequence (5′- > 3′)
EGFP-EcoRI-fwd	ggcatcatgaattcgtttgtgaacgacattttcgagc
EGFP-RKKRKKK-NoLS-XbaI-rev	ttaatctagattacttcttttttcgcttctttctcttgtacagctcgtccatgccgagagtg
EGFP-R9-NoLS-XbaI-rev	ttaatctagattatctccttctccttctccttctccttctcttgtacagctcgtccatgccgagagtg
EGFP-WRRQARFK-NoLS-XbaI-rev	ttaatctagattacttaaacctggcttggcgtctccacttgtacagctcgtccatgccgagagtg
Fr1_for_Kozak_H2B	gagaccccggatcggggtacccaccatgcctccgaaaactag
Fr1_rev_pitch	cctggaaatcggtggcgaccggtgg
Fr2_for_pitch	ggtcgccaccgatttccaggaggcgccg
Fr2_rev_pitch	tgctcaccatatccgagttggccacctg
Fr3_for_pitch_EGFP	caactcggatatggtgagcaagggcgag
Fr2_rev_EGFP_NS3	gatcattgcccttgtacagctcgtccatgc
Fr3_for_NS3	gctgtacaagggcaatgatccggcgg
Fr3_rev_NS3	gcttaccttcgaagggccctctagattagtgctcgtccaggtgc
Fr1_for_Non3	gagaccccggatcggggtacccaccatgtcgcttttacgcatcag
Fr1_rev_Non3_H2B	tcggaggcatgagttgcggctccctgg
Fr2_for_Non3_H2B	gccgcaactcatgcctccgaaaactagtggaaag
Fr1_for_NS1	gagaccccggatcggggtacccaccatggctttaaaaaggttgaagacc
Fr1_rev_NS1	tcggaggcatgaagggacagatgtttgggatctg
Fr2_for_NS1_H2B	ctgtcccttcatgcctccgaaaactagtggaaag
Fr1_rev_Non3	tacagatcctcggtggcgaccggtgg
Fr2_for_Non3	ggtcgccaccgaggatctgtacaaacaggcacg
Fr2_rev_Non3	tgctcaccatggcgtctgtgctaatgttcttct
Fr3_for_Non3_EGFP	cacagacgccatggtgagcaagggcgag
Fr2_rev_EGFP_mod	tctgaccaatcttgtacagctcgtccatgc
Fr3_for_mod	gctgtacaagattggtcagacccgcg
Fr3_rev_mod	gcttaccttcgaagggccctctagattatttaccaccattctcttgtgcc

## Data Availability

Data is contained within the article.
